# Evolving paradigms in cardiogenic shock care

**DOI:** 10.18632/aging.102075

**Published:** 2019-07-02

**Authors:** Behnam N. Tehrani, Carolyn M. Rosner, Wayne B. Batchelor

**Affiliations:** 1 Cardiac Catheterization Laboratory, INOVA Heart and Vascular Institute, Falls Church, Virginia 22042, USA

**Keywords:** cardiogenic shock, percutaneous mechanical circulatory support

The findings of the landmark Should we Emergently Revascularize Occluded Coronaries for Cardiogenic Shock (SHOCK) Trial significantly altered our treatment approach to patients with acute myocardial infarction (AMI) complicated by cardiogenic shock (CS) [1]. With a number needed to treat of less than eight to save one life, early revascularization was irrefutably shown to afford a substantial survival benefit to patients afflicted with a syndrome previously associated with an 80% mortality rate. Despite further advances in AMI treatment, including the implementation of regionalized systems of care, adoption of transradial arterial access and the advent of percutaneous mechanical circulatory support (PMCS) devices, 30-day mortality in CS has stagnated at 40-50% for two decades [2]. These suboptimal outcomes have been seen across all patient populations, including adults ≥ 75 years of age with ST-elevation myocardial infarction (STEMI), who are undergoing higher rates of invasive therapies, despite having a significant burden of co-morbidities, including a >10% rate of CS [3]. Notwithstanding difficulties with constructing adequately powered randomized controlled trials (RCT), we are witnessing a resurgence of inquiry surrounding CS care, fueled by clinical research using observational registries to identify innovative ways to stem the current tide of morbidity and mortality.

In response to the absence of clear guidelines for the care of patients with this hemodynamically complex and multifactorial disease state, the Society for Cardiovascular Angiography and Interventions (SCAI) recently released a consensus statement on the classification of CS [4]. A multidisciplinary working group consisting of thought leaders from interventional cardiology, advanced heart failure, critical care, emergency medicine and nursing proposed a five stage classification schema, spanning from patients “At Risk” for CS to those in “Extremis”, typically in frank circulatory collapse and requiring multiple life-saving interventions. Building on the established Interagency Registry for Mechanically Assisted Circulatory Support (INTERMACS) nomenclature system, the SCAI authors elegantly ascribed three core domains to each CS stage which are straightforward and readily garnered by all team members involved in the care of the CS patient: physical exam findings, biochemical markers and hemodynamic assessments. The development of a universal language around disease severity is a seminal achievement towards improving patient identification and treatment and also facilitating trial designs to evaluate current and future medical and device-based therapies for patients with this syndrome.

The discipline of structural heart disease has embraced a model of multidisciplinary team- based care, known as the Heart Team, to provide collaborative and patient-centered care for patients with valvular heart disease. Despite clinical precedent for such an approach with other complex cardiac conditions, the management of CS has remained largely fragmented with widely disparate practice patterns for decades [2]. Despite the superior hemodynamic benefit associated with axial and centrifugal flow PMCS devices compared to the intra-aortic balloon pump, their survival benefit has yet to be borne out in RCTs, in part due to lac k of power and inconsistencies in patient selection. However, there is now increasing evidence from single and multi-center observational registries suggesting that the implementation of cross-disciplinary shock teams utilizing protocol-driven care that emphasizes mandatory and serial hemodynamic assessments is feasible and may also improve survival in patients with CS due to both AMI and acute decompensated heart failure [5,6]. Using validated markers predictive of in-hospital mortality, patients can be readily risk stratified and offered therapies that fulfill all of the treatment objectives of the hemodynamic support equation, including the evolving science of ventricular unloading where future studies will reexamine the current door-to-balloon metric in AMI care [7]. These studies also suggest that CS care may be optimized with the development of tiered and regionalized systems, similar to those implemented for the treatment of AMI 15 years ago, where patients are quickly triaged and transferred to tertiary and quaternary cardiac intensive care units with dedicated staffing models and equipped to provide full spectrum and longitudinal care.

**Figure 1:**
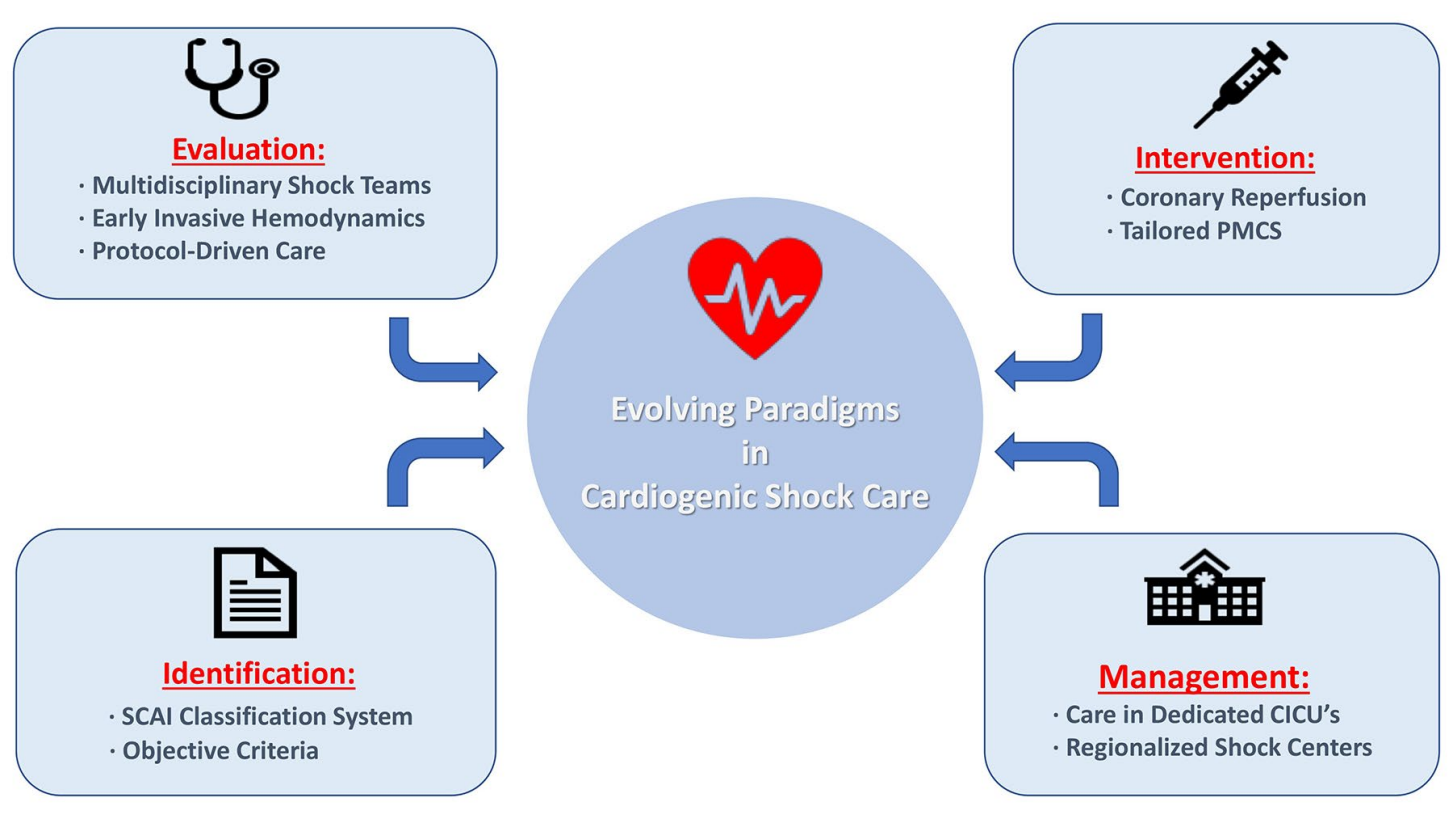
**Cardiogenic shock care delivery models emanating from population-based registries.** Abbreviations: CICU = cardiac intensive care unit; PMCS = percutaneous mechanical circulatory support; SCAI = Society for Cardiovascular Angiography and Interventions.

Twenty years after the SHOCK Trial, we are now at an inflection point in acute cardiovascular care. Using population-based registries, clinicians and researchers are coming together to identify innovative ways to improve recognition and treatment of CS across the entire disease spectrum (Figure 1). Therefore, it is incumbent upon us to use this data in a meaningful way and construct pragmatic trials designed to ultimately inform clinical guidelines and advance the care of our patients.
